# Oral pyruvate prevents high-intensity interval exercise-induced metabolic acidosis in rats by promoting lactate dehydrogenase reaction

**DOI:** 10.3389/fnut.2023.1096986

**Published:** 2023-04-06

**Authors:** Kaixuan Che, Yanping Yang, Jun Zhang, Lin Feng, Yan Xie, Qinlong Li, Junqiang Qiu

**Affiliations:** ^1^Department of Exercise Biochemistry, Exercise Science School, Beijing Sport University, Beijing, China; ^2^Department of Exercise Physiology, Exercise Science School, Beijing Sport University, Beijing, China; ^3^Beijing Sports Nutrition Engineering Research Center, Beijing, China

**Keywords:** metabolic acidosis, high-intensity interval exercise, lactate dehydrogenase reaction, glycolysis, redox

## Abstract

**Introduction:**

There is no denying the clinical benefits of exogenous pyruvate in the treatment of pathological metabolic acidosis. However, whether it can prevent exercise physiological metabolic acidosis, delay the occurrence of exercise fatigue, and improve the beneficial effects of exercise and its internal mechanism remain unclear.

**Methods:**

We randomly divided 24 male SD rats into 3 groups: one group was a control without exercise (CC, *n* = 8), and the other two groups were supplemented with 616 mg/kg/day pyruvate (EP, *n* = 8) or distilled water of equal volume (EC, *n* = 8). These groups completed acute high-intensity interval exercise (HIIE) after 7 days of supplementation. The acid metabolism variables were measured immediately after exercise including blood pH (pH_e_), base excess (BE), HCO_3_^−^, blood lactic acid and skeletal muscle pH (pH_i_). The redox state was determined by measuring the oxidized coenzyme I/reduced coenzyme I (nicotinamide adenine dinucleotide [NAD^+^]/reduced NAD^+^ [NADH]) ratio and lactate/pyruvate (L/P) ratio. In addition, the activities of lactate dehydrogenase A (LDHA), hexokinase (HK), phosphofructokinase (PFK) and pyruvate kinase (PK) were determined by ELISA.

**Results:**

Pyruvate supplementation significantly reversed the decrease of pHe, BE, HCO_3_^−^ and pH_i_ values after HIIE (*p* < 0.001), while significantly increased the activities of LDHA (*p* = 0.048), HK (*p* = 0.006), and PFK (*p* = 0.047). Compared with the CC, the NAD+/NADH (*p* = 0.008) ratio and the activities of LDHA (*p* = 0.002), HK (*p* < 0.001), PFK (*p* < 0.001), and PK (*p* = 0.006) were significantly improved in EP group.

**Discussion:**

This study provides compelling evidence that oral pyruvate attenuates HIIE-induced intracellular and extracellular acidification, possibly due to increased activity of LDHA, which promotes the absorption of H^+^ in the LDH reaction. The beneficial effects of improving the redox state and glycolysis rate were also shown. Our results suggest that pyruvate can be used as an oral nutritional supplement to buffer HIIE induced metabolic acidosis.

## 1. Introduction

Metabolic acidosis is an acid–base balance disorder caused by the lack of alkaline substances and/or the excess of acidic substances (except CO_2_) in blood (1). During high-intensity exercise, H^+^ released by adenosine triphosphate (ATP) hydrolysis cannot be cleared out of cells in time, which is the main cause of metabolic acidosis (2, 3). Therefore, exercise-induced metabolic acidosis is due to a decrease in mitochondrial oxidative phosphorylation for energy supply under relative hypoxia induced by high-intensity exercise, which relies on anaerobic glycolysis for energy supply. This phenomenon leads to the accumulation of H^+^ and lactic acid in tissues and the reduction of the blood buffer substance HCO_3_^−^ (3, 4). Previous studies have shown that high-intensity interval exercise (HIIE) can induce metabolic acidosis in a short time (5–7). High-intensity exercise can increase ATP demand rate by 1,000 times relative to that in the resting state. This process relies on glycolytic ATP hydrolysis for energy production, and the generated H^+^ cannot be completely removed during the relief interval. With the repetition of exercise bouts, metabolic acidosis is induced. Acidosis in this exercise mode is a unique form of metabolic change (8, 9). In addition, this type of exercise is an important constitute of many sports that require intermittent explosiveness (especially team sports). on the other hand, the inhibiting effects of low pH on Ca^2+^ sensitivity, velocity and peak power in HIIE support the conclusion that acidosis is an important fatiguing agent (10). As such, exploring the approach to relieve metabolic acidosis in HIIE has guiding significance for delaying exercise fatigue in competitive sports.

Pyruvate is a key intermediate in the body’s metabolic pathway. Furthermore, it can be used as a new alkaline buffer in clinical practice to exert superior biomedical properties through exogenous supplementation (11–13). In recent years, studies on clinical trials have verified the corrective effect of exogenous pyruvate on metabolic acidosis, which can relieve blood acidity by combining weak base anions with H^+^ (14, 15). However, the effect of pyruvate on metabolic acidosis is not only the result of its chemical buffering ability as an alkalizing agent but may also be mainly through its biological properties to consume excess H^+^, thus effectively avoiding or correcting metabolic acidosis (16). Notably, the lactate dehydrogenase (LDH) reduction reaction has a unique advantage in the pathway of pyruvate-correcting metabolic acidosis. First, pyruvate as a sole substrate, can be spontaneously converted into lactate by LDH, and the carboxylate anion of each exogenous pyruvate absorbs an H^+^ from the cytoplasmic hydrogen pool in this process. The reduction of pyruvate is a systemic alkalinizing reaction with H^+^ consumption, raising the pH in hypoxia and even in anoxia (3, 17). Another important function of the LDH reaction is the production of cytosolic nicotinamide adenine dinucleotide (NAD^+^), which supports the NAD^+^ substrate demand of the glyceraldehyde 3-phosphate dehydrogenase reaction. This process then improves the maintenance of the cytosolic redox potential (NAD^+^/reduced NAD^+^ [NADH]) and supports the continuous substrate flux through phase two of glycolysis, which allows constant ATP regeneration from glycolysis (3, 14) (see Supplementary Material).

At present, the effect of short-term pre-supplementation of pyruvate on exercise-induced metabolic acidosis has been scarcely reported. It was preliminarily confirmed in human experiments in our laboratory that pyruvate supplementation may accelerate restoration of the acid–base balance, but its internal mechanism remains unclear (18). Moreover, whether oral pyruvate can alleviate acidosis by improving the LDH reaction needs further verification. Available evidence has shown that exogenous pyruvate can treat pathological metabolic acidosis (e.g., hypoxic metabolic acidosis associated with lethal hemorrhagic shock) in rats, raise intracellular pH (pH_i_) and improve glycolysis in hypoxic, ischemic or even anoxic conditions by sustaining the NAD^+^/NADH with the LDH reaction (14). Thus, we hypothesized that pyruvate pre-supplementation can effectively prevent HIIE-induced metabolic acidosis, mitigate acidification of blood and skeletal muscle in rats by accelerating H^+^ consumption in LDH reaction and provide beneficial effects in improving redox state and glycolysis metabolism. Acid metabolism variables, redox status, lactate dehydrogenase A (LDHA) enzyme activity and rate-limiting glycolytic enzyme activities are determined in rat blood or skeletal muscle.

## 2. Materials and methods

### 2.1. Animals and groups

A total of 24 healthy specific pathogen-free (SPF) male SD rats (weight: 316.8 ± 11.7 g, age: 8 weeks) were obtained from the Beijing Vital River Laboratory Animal Technology Co., Ltd., with license No. SCXK (Beijing) 2019–0010. All animals were raised in separate cages, with four rats in each cage provided with a free diet (adaptive feeding for 3 days) at a room temperature of 20°C–25°C and a relative humidity of 50–70%. According to the random principle, the animals were divided into control non-exercise group (CC, *n* = 8), control HIIE group (EC, *n* = 8) and pyruvate pre-supplemented HIIE group (EP, *n* = 8). This study was approved by the Institutional Review Board of Beijing Sport University (approval no. 2020057H) and conformed to the Regulations for the Administration of Affairs Concerning Experimental Animals published by the State Science and Technology Commission in China.

### 2.2. Experimental testing overview

**Figure 1:**
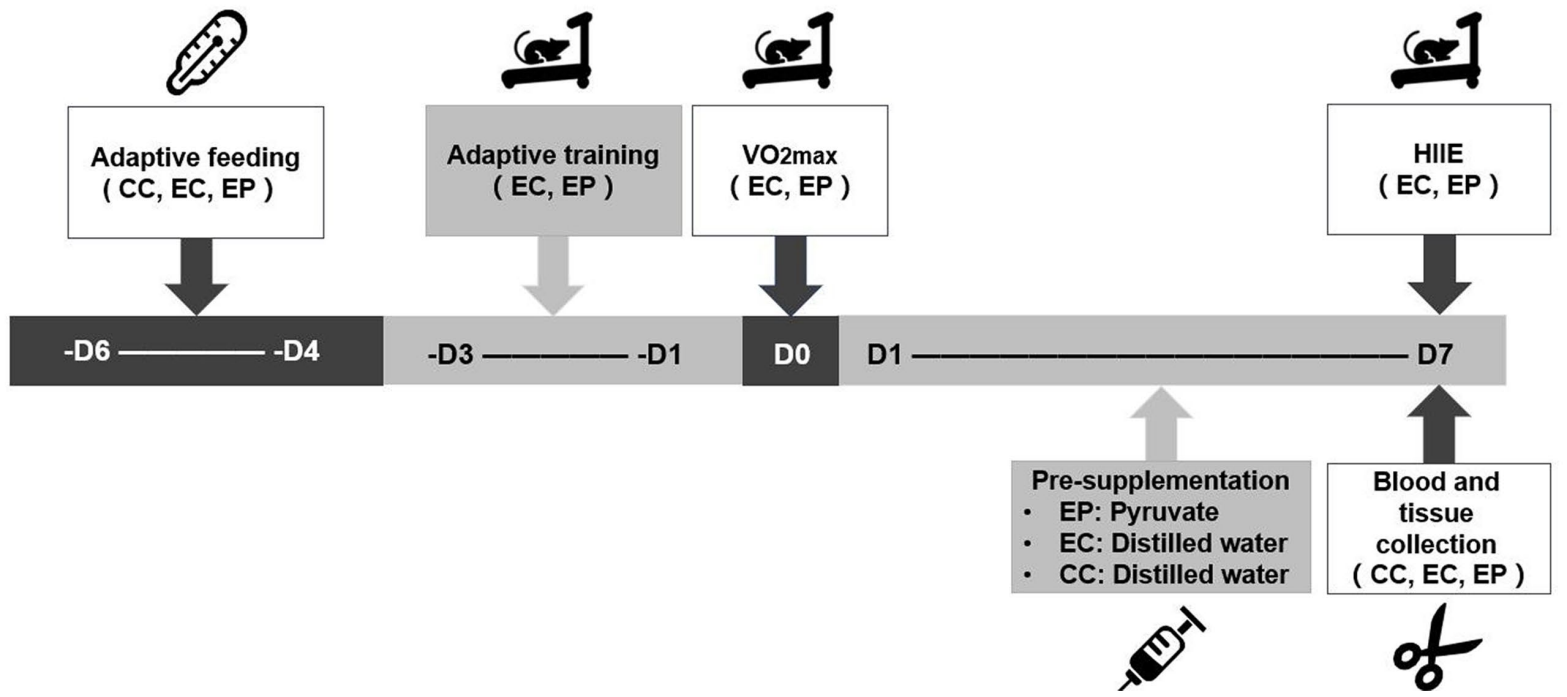
Experimental testing overview. VO_2max_ was completed prior to the experiment to determine the load intensity of the high-intensity interval exercise (HIIE). A 7-day pyruvate/distilled water supplementation was then completed prior to HIIE.

The rats in the EP and EC completed a three-day treadmill adaptive training and a one-day maximal oxygen uptake (VO_2max_) test prior to the experiment. The EP then completed 7 days of pyruvate supplementation, whereas the CC and EC completed 7 days of distilled water supplementation. An hour after the end of pyruvate/distilled water supplementation on the 7th day, the rats in the EP and EC completed the HIIE. Immediately after the HIIE, the abdominal aortic blood and extensor digitorum longus (EDL) muscle of the rats were collected to determine blood acid metabolism variables, including blood pH (extracellular pH, pH_e_), base excess (BE), HCO_3_^−^ content and blood lactic acid (LAC) level. Skeletal muscle pH (pH_i_) and LDHA, hexokinase (HK), phosphofructokinase (PFK) and pyruvate kinase (PK) enzyme activities, as well as NAD^+^/NADH and lactate/pyruvate (L/P) ratios, were measured in the EDL muscle tissue (Figure 1).

### 2.3. Experimental protocol

#### 2.3.1. Supplementary protocol

Sodium pyruvate was dissolved in distilled water and administered to the EP at a dose of 616 mg/kg body weight (b.w.) by gastric gavage daily for 7 days (19, 20). The supplementary protocol was similar to previously reported protocol by our laboratory (18). Similarly, the rats in the control group received the same amount of distilled water for the same duration.

#### 2.3.2. Exercise protocol

Treadmill adaptive training: On the first day, the rats in the HIIE group ran for 5 min on a treadmill at a speed of 16 m/min and a slope of 10°. On the second and third days, the speed and slope of the treadmill remained unchanged, but the running time was increased by 5 min.

VO_2max_ test: The protocol began with a four-minute warm-up at 5 m/min. The starting speed for the test was 10 m/min, followed by three-minute incremental stages, with a 5 m/min increase in speed at each stage, and the gradient remained unchanged at 10° throughout the process until the rats were exhausted (21). During the test, ventilation samples were collected continuously using an animal indirect calorimetry system (CLAMS, Columbus, Canada), which was calibrated prior to each test. VO_2_ was recorded every 30 s and used to calculate VO_2max_.

HIIE: According to the VO_2max_, HIIE was completed at a running speed of 110% VO_2max_ intensity (Speed_HIIE_), with 1 min of exercise and 1 min of rest and a total of 13 bouts (5). The VO_2max_ and Speed_HIIE_ are shown in Table 1.

**Table 1:** VO_2max_ and Speed_HIIE_ in HIIE rats($\bar{x}\pm$ SD).

	VO_2max_ (ml/kg·h^−1^)	Speed_HIIE_ (m/min)
EC	3735.92 ± 329.42	41.25 ± 5.77
EP	3807.48 ± 466.36	41.23 ± 4.61

VO_2max_, maximal oxygen uptake; Speed_HIIE_, 110% VO_2max_ intensity.

### 2.4. Blood and tissue collection

Immediately after HIIE, the rats were anesthetized intraperitoneally with 2% pentobarbital sodium injection (50 mg/kg b.w.). Abdominal aortic blood was collected from exercised and non-exercised rats for arterial blood gas analysis, in which pH_e_ < 7.35, HCO_3_^−^ < 22 mM and LAC > 5 mM of the EC were judged as exercise-induced metabolic acidosis. The EDL muscle from the hindlimb of every rat was then rapidly excised, and the fresh tissue on one side was stored on ice for immediate NAD^+^/NADH ratio determination and the other side was stored at −80°C until further biochemical analysis of other indicators.

### 2.5. Samples analysis

#### 2.5.1. Determination of acid metabolic variables

The blood acid metabolism variables of the collected 0.5–1 ml arterial blood were measured using an automatic blood gas analyzer (ABL90 OSM, RADIOMETER, Denmark), and the pH_e_, BE (mM), HCO_3_^−^ (mM) and LAC values (mM) were recorded.

For the determination of pH_i_ in skeletal muscle, 80 mg of muscle tissue was homogenized in 800 μl of non-buffered solution (145 mM NaCl, 10 mM KCl, and 5 mM NaF). The pH_i_ of the homogenate was measured using a pH meter (FE28-Standard, METTLER TOLEDO, Switzerland).

#### 2.5.2. Assessment of the redox state

The NAD^+^/NADH in the muscle samples was measured with the WST-8, as described by Chamchoy et al. (22) using the NAD^+^/NADH detection kit (Cat#S0175, Beyotime Biotechnology Co., Ltd., Shanghai, China). Muscle tissue was homogenized in the NAD^+^/NADH extract and centrifuged at 12,000 rpm for 10 min at 4°C. The collected supernatant was used to quantify NADH and NAD_total_ (total NAD^+^ and NADH) at 450 nm by spectrophotometer (Synergy, BioTek, United States), and the NAD^+^/NADH ratio was calculated.

Lactate and pyruvate were relatively quantified by high-performance liquid chromatography-mass spectrometry. Approximately 50 mg of muscle tissue was homogenized in 200 μl of methanol/acetonitrile/water mixture (2:2:1) and centrifuged at 14,000 rpm for 20 min at 4°C. The supernatant was transferred to an autosampler plate for analysis. Samples were separated by high-performance liquid chromatography (Ultimate 3,000, Thermo Fisher Scientific, United States) and analyzed by mass spectrometer (Q-Exactive, Thermo Fisher Scientific, United States). The chromatographic peak area and retention time were measured, lactate and pyruvate were quantified, and their ratio (L/P) was calculated.

#### 2.5.3. Measurement of glycolytic key enzymes activity

For the determination of LDHA, HK, PFK and PK activities, the muscle samples were homogenized in 10 mM PBS buffer (pH = 7.4) and centrifuged at 5000 rpm for 10 min at 4°C. The supernatants were then carefully collected. The relevant enzyme activity in muscles was detected using standard methods for rat LDHA, HK, PFK, and PK using an ELISA kit (23), and the kits were provided by Jianglai Technology Co., Ltd. (Cat#JL48282, JL11496, JL21140, JL20916, Jianglai, Shanghai, China). The standard curve was made on enzyme coated plate with 50 μl different concentrations of a standard substance. 50 μl of sample diluent was added to the enzyme coated plate, and then 50 μl of the sample to be tested was added. 100 μl of enzyme-labeled reagent was added into each well. The mixture was incubated at 37°C for 60 min, then washed 5 times and left to dry. Chromogenic agent solutions A and B (50 μl each) were added and incubated at room temperature for 15 min in the dark. After adding 50 μl of the termination solution, the OD values of each pore were measured by an Enzyme labeling instrument (Synergy, BioTek, United States) at 450 nm.

### 2.6. Statistical analyses

Statistical analyses were performed using SPSS 22.0. For the data with normal distribution, an one-way analysis of variance (ANOVA) with Tukey *post hoc* test was used to evaluate the significant differences among the three groups, and the value was reported as $\bar{x}\pm$ SD. For the data with non-normal distribution, the Kruskal–Wails test was used to evaluate the significant difference among the three groups, and the value was reported as the median (interquartile range). Significance was accepted at *p* < 0.05.

## 3. Results

### 3.1. Effect of pyruvate pre-supplementation on pH_e_, Be, HCO_3_^−^, LAC, pH_i_ values in HIIE rats

**Figure 2:**
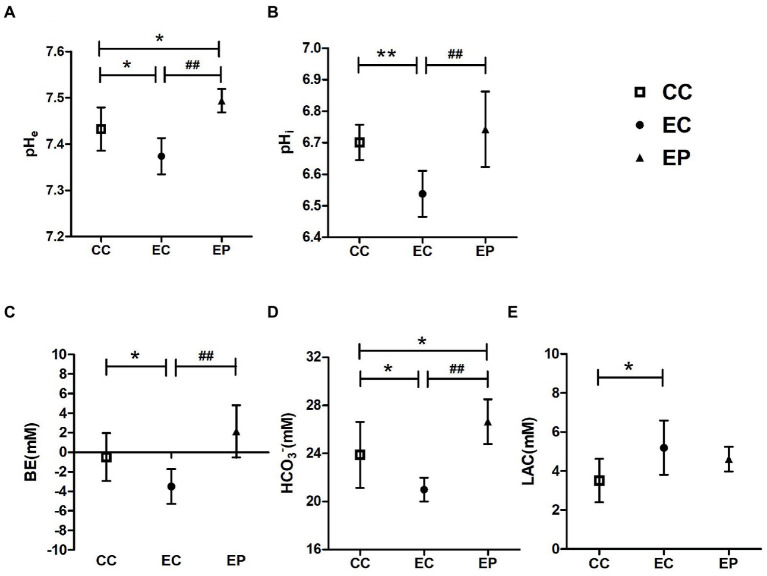
Effect of exogenous pyruvate pre-supplementation on **(A)** blood pH (pH_e_), **(B)** muscle pH (pH_i_), **(C)** base excess (BE), **(D)** HCO_3_^−^, **(E)** lactic acid (LAC) levels in high-intensity interval exercise rats. *Significantly different to control non-exercise (CC) group (*p* < 0.05). **Significantly different to control non-exercise (CC) group (*p* < 0.01). ^##^Significantly different to control HIIE (EC) group (*p* < 0.01).

HIIE-induced acidosis in blood and muscle tissue were estimated by measuring the levels of pH_e_, BE, HCO_3_^−^, LAC, and pH_i_, respectively. HIIE significantly decreased pH_e_ (*p* = 0.015; Figure 2A), BE (*p* = 0.044; Figure 2C), HCO_3_^−^ (*p* = 0.023; Figure 2D), pH_i_ (*p* = 0.003; Figure 2B)levels and increased LAC (*p* = 0.014; Figure 2E) levels in control group, which indicates increased acidification in blood and muscle tissue. Exercise-induced reductions in pH_e_, BE, HCO_3_^−^, pHi levels were significantly reversed in the EP (*p* < 0.001; Figures 2A–D). The pH_e_ (*p* = 0.011; Figure 2A) and HCO_3_^−^ (*p* = 0.030; Figure 2D) were significantly higher in the EP compared to the CC. However, no significant change in blood LAC levels was observed with pyruvate pre-treatment (*p* > 0.05; Figure 2E).

### 3.2. Effect of pyruvate pre-supplementation on NAD^+^/NADH and L/P ratio in HIIE rats

**Figure 3:**
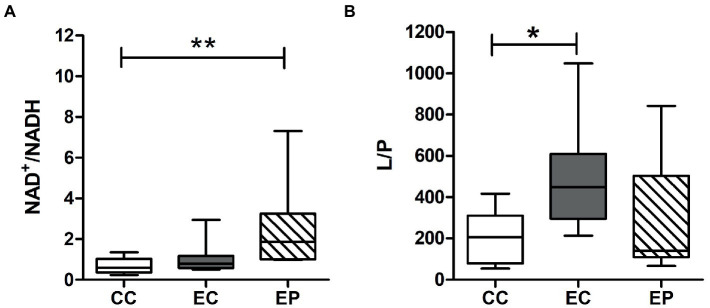
Effect of exogenous pyruvate pre-supplementation on **(A)** nicotinamide adenine dinucleotide (NAD^+^)/reduced NAD^+^ (NADH) ratio and **(B)** lactate/pyruvate (L/P) ratio in high-intensity interval exercise rats. *Significantly different to control non-exercise (CC) group (*p* < 0.05). **Significantly different to control non-exercise (CC) group (*p* < 0.01).

Figure 3 indicated redox status change in the studied groups. Skeletal muscle L/P ratio was significantly increased (*p* = 0.049) after HIIE in control group, indicating a decrease in the redox status. However, this change was not found in pyruvate pre-treated exercised rats (Figure 3B). In contrast to the L/P results, the NAD^+^/NADH ratio was not influenced by HIIE in the control group but increased in the EP (Figure 3A). This increase was statistically significant (*p* = 0.008) compared to the CC (Figure 3A), which indicates an increase of skeletal muscle redox status in pyruvate pre-treated exercised rats.

### 3.3. Effect of pyruvate pre-supplementation on LDHA, HK, PFK and PK activities in HIIE rats

**Figure 4:**
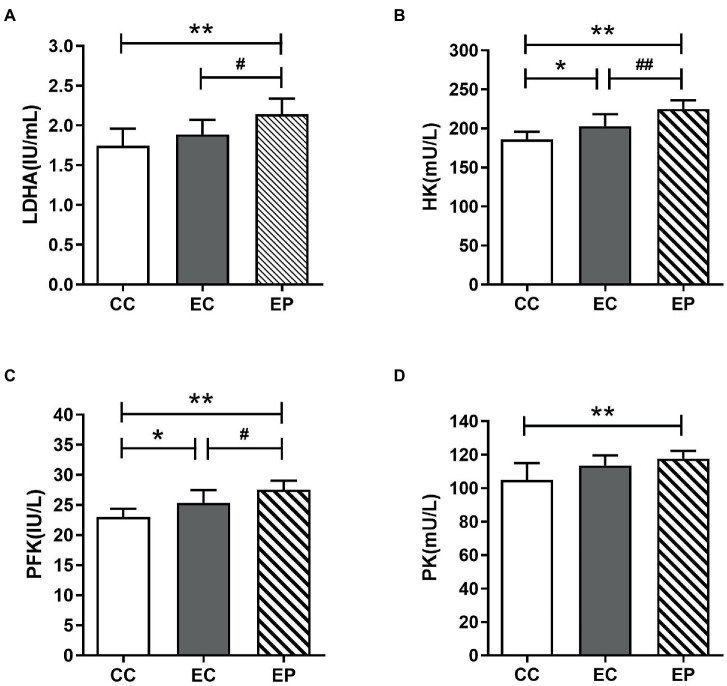
Effect of exogenous pyruvate pre-supplementation on glycolytic key enzymes activity in high-intensity interval exercise rats. **(A)** Lactate dehydrogenase A (LDHA) concentration. **(B)** Hexokinase (HK) concentration. **(C)** Phosphofructokinase (PFK) concentration. **(D)** Pyruvate kinase (PK) concentration.*Significantly different to control non-exercise (CC) group (*p* < 0.05). **Significantly different to control non-exercise (CC) group (*p* < 0.01). ^#^Significantly different to control HIIE (EC) group (*p* < 0.05). ^##^Significantly different to control HIIE (EC) group (*p* < 0.01).

The changes in HK, PFK PK, and LDHA activities are shown in Figure 4, which indicate the rate of glycolysis and LDH reduction reaction, respectively. HIIE increased the LDHA, HK, PFK, and PK activities in the control group, with statistically significant increases in HK (*p* = 0.036; Figure 4B) and PFK (*p* = 0.030; Figure 4C). The EP further increased LDHA, HK, PFK and PK activities. The increases in LDHA (*p* = 0.048; Figure 4A), HK (*p* = 0.006; Figure 4B) and PFK (*p* = 0.047; Figure 4C) activities were statistically significant compared to that in the EC, and these values were significantly higher compared to that in the CC. Similarly, PK was also increased significantly compared to that in the CC (*p* = 0.006; Figure 4D).

## 4. Discussion

The main finding of this study is that pyruvate taken orally for 7 days can promote the absorption of H^+^ in the LDH reaction, further preventing exercise-induced metabolic acidosis. Consistent with this finding, the decreased pH_i_, pH_e_, BE, and HCO_3_^−^ after HIIE was reversed, and LDHA activity was significantly increased in the EP. In addition, pyruvate pre-treated skeletal muscle redox status and glycolytic enzyme activity were improved. These results provide compelling evidence that oral pyruvate can prevent HIIE-induced metabolic acidosis in rats by promoting the LDH response, while simultaneously improving the redox state and glycolysis rate.

Arterial blood gas analysis can assist in the assessment of the acid–base balance in clinical practice. Metabolic acidosis is diagnosed when the parameters most frequently used—pH (7.35–7.45), BE (−2 ± 2 mM) and HCO_3_^−^ (22–26 mM)—fluctuate below the standard range (1). In current human experiments that performed different combinations of HIIE in 4–6 bouts, with each bout approaching or exceeding the maximum intensity exercise from 30 s to 1 min, the body is acidified to varying degrees, resulting in increased lactate and HCO_3_^−^ levels and decreased pH_i_ (muscle tissue)/pH_e_ (blood) levels (5–7, 24). In animal experiments, it was also found that performing a six–bout high-intensity exercise for 2 min each at 80% peak speed with a 1-min interval, the pH_i_ has been decreased to 6.6 (6.54–6.66) (25). In our study, pH_e_, pH_i_, BE, HCO_3_^−^ levels were significantly decreased, and LAC level was significantly increased after HIIE compared with the CC. Although the post-exercise blood pH (pH_e_ = 7.37 ± 0.04) slightly exceeded the lower limit of the clinical standard range (1), it was still lower than the normal range of blood pH in rats reported in previous studies (26), and other blood gas indexes all satisfied the criteria for metabolic acidosis. Skeletal muscle pH also decreased to approximately 6.5, which is consistent with the results reported by Hedges et al. (27). From the abovementioned findings, the exercise mode of HIIE in this study successfully induced metabolic acidosis.

Pyruvate can effectively correct metabolic acidosis, an effect which has been demonstrated in many preliminary experiments. As early as 1999, Mongan et al. first highlighted the “systemic alkalization” induced by intravenous pyruvate (28). It was found that pyruvate injection significantly increased pH, BE, and HCO_3_^−^ levels and prolonged survival time in a swine hemorrhagic shock model. The phenomena in which pyruvate increased pH and BE values in swine and rats are then discovered one after the other on pathological metabolic acidosis (29–31); however, no additional studies have investigated the effect of oral pyruvate on exercise-induced metabolic acidosis, especially on skeletal muscle pH, in exercise rats. Our data showed that oral pyruvate significantly reversed the decline in pH_e_, BE, HCO_3_^−^, and pH_i_ values after HIIE in rats. This is explained by the more H^+^ consumption *via* the LDHA reaction following the pyruvate supplementation (as described below) and thus alkaline substances in blood were preserved. The preliminary results presented within this study suggest that oral pyruvate is effective in alleviating exercise-induced metabolic acidosis, similar to that found in models of pathological metabolic acidosis (19), and alkalizing both intracellular and extracellular environments.

Lactate dehydrogenase A activity in the current investigation was significantly elevated in the EP compared with that in the EC. To our knowledge, this report is the first to demonstrate the significant effect of pyruvate on up-regulating LDHA activity in skeletal muscle, aiming to explore the possible mechanism for acidosis alleviation. As a marker enzyme of anaerobic metabolism, LDHA can promote LDH reaction in the direction of consuming H^+^, and when the oxygen supply is limited, the regenerated NAD^+^ can further maintain glycolysis (32). Notably, NAD^+^, as a cofactor for several enzymatic activities, can provide the need to maintain continuous muscle activity, and the NAD^+^/NADH ratio is a key factor that reflects cellular redox status and regulates cellular metabolism (33). In this study, increased LDHA activity in pyruvate-treated exercised rats may contribute to the elevation of NAD^+^/NADH ratio. Pyruvate can convert pyruvate to lactate through the coupling of LDH to NADH oxidation, while elevating NAD^+^/NADH ratio and accelerating proton depletion (14, 34). However, there was no significant increase in blood LAC level in the current study, which could be attributed to lactate transport and further absorption and utilization by other tissues. As previous studies have concluded, when lactate flux increases, monocarboxylate transporters (MCTs) will promote lactate anion to co-cross the membrane barrier with H^+^(1,1 molecular ratio), and continue to act as metabolic substrates and key signaling molecules in skeletal muscle, heart, brain, and other tissues (35–37). Therefore, we hypothesize that pyruvate may further improve the lactate transport rate while accelerating the lactate production, so there was no accumulation of LAC, which provides the direction for future studies to further explore the effect of pyruvate on lactate transport. Furthermore, pyruvate pre-treatment reduces L/P ratio, and the stabilization of this ratio suggests that a large NAD^+^/NADH ratio is preserved with decreasing L/P ratio (38). The trend toward increased NAD^+^/NADH ratio and reduced L/P ratio in animal models of metabolic acidosis after pyruvate pre-treatment, as observed in the previous studies, may be indicative of pyruvate that can increase the redox potential in multiple tissues (38, 39). In the current study, no significant differences in NAD^+^/NADH and L/P were observed between the EP and EC. This may be partly explained by the fact that NAD^+^ is easily degraded and unstable *in vitro* due to the influence of temperature or acid–base environment, so it is easy to transform with NADH. Similarly, lactate concentration will change greatly with the delay of time, while pyruvate concentration remains relatively constant, and the L/P ratio will also fluctuate (40–42). However, compared with the quiet control group, EP showed a significant increase in NAD^+^/NADH ratio, and EC exhibited a significant increase in L/P ratio. Given that the increase in L/P ratio after exercise-induced acidosis indicated impaired redox status, which was mitigated by pyruvate pre-treatment with no difference from the quiet status, and accompanied by an increase in the NAD^+^/NADH ratio, the results of the current pilot study may provide a preliminary indication that the redox status may be improved following pyruvate pre-treatment. Further studies on the effects of a pyruvate pre-treatment on NAD^+^/NADH ratio and L/P ratio are required however, given the small sample size used in the current study. The present study adds to the current knowledge that the up-regulation of LDHA activity may be the key to promoting the LDH reaction after pyruvate supplementation, which contributes to the accelerated depletion of H^+^, alleviates the phenomenon of exercise-induced metabolic acidosis, and potentially improves the redox state.

The key rate-limiting enzymes in the glycolytic pathway include HK, PFK and PK. Previous studies have shown that HK (43), PFK (44), and PK (45) increase skeletal muscle activity after intense exercise in rats. Both HK and PFK activities showed similar responses after acute HIIE. However, our data showed no significant increase in PK activity after HIIE in the control group. This difference may be due to a decrease in pH of muscle tissue, inhibiting its activity or different movement patterns. Pyruvate enhances NAD^+^/NADH ratio simultaneously induced in the pyruvate reductive reaction, promoting the regular glycolysis pathway (34, 46). Increases in HK, PFK, and PK activities after pyruvate supplementation in exercised rats are found to be regulated by hypoxia-inducible factor-1α (HIF-1α). Some previous studies have reported that pyruvate inhibits HIF-1α degradation and enhances HIF-1α activity. HIF-1α is a key transcription factor that upregulates a series of downstream genes involved in glucose metabolism and promotes the expression of glucose metabolization-related transporters and enzymes. Such as the expression of glucose transporters, phosphoglycerate kinase 1 (PGK1), pyruvate dehydrogenase kinase 1 (PDK1), LDHA and glycolytic enzymes such as HK1, HK2, PFK1, PFK2 and PKM2 (16, 47, 48). The up-regulation of these proteins is conducive to the increase of glycolysis rate and the consumption of H^+^. In our study, oral pyruvate upregulated the activity of three key rate-limiting enzymes in the glycolytic pathway in HIIE rats, which may be partly responsible for the increased glycolytic rate. Given the limitations of the current study on interpretation at the protein or gene level, further research is warranted, especially on the regulatory mechanisms of HIF-1α.

## 5. Conclusion

The results of the study provide strong evidence that 7-day oral pyruvate can effectively attenuate HIIE-induced intracellular and extracellular acidification, which may be explained by increased LDHA activity, thus promoting the LDH reaction to absorb H^+^. The beneficial effect of pyruvate is also explained, in part, by the improvement of redox state and the increase of glycolysis rate in skeletal muscle. The findings of the study suggest that pyruvate can be used to design nutraceutical supplements aimed to preserve homeostasis against HIIE-induced metabolic acidosis, which may be important in preventing exercise fatigue and ensuring the energy supply during sports competitions and its rapid recovery afterwards.

## Data availability statement

The raw data supporting the conclusions of this article will be made available by the authors, without undue reservation.

## Ethics statement

The animal study was reviewed and approved by Institutional Review Board of Beijing Sport University (BSU IRB) (No. 2020057H).

## Author contributions

KC was responsible for the operation of the experiment, data collection, data interpretation, writing and revision of the manuscript, under the direction and assistance of JQ who assisted with each step and completion of the manuscript. YY, JZ, and YX assisted in the completion of the experiment. LF and QL assisted in the revision of the manuscript. All authors contributed to the article and approved the submitted version.

## Funding

Funding was provided by the Herbalife Winter Sports Nutrition Research Fund (Nos. KBL2019003 and KBL2021007) and the Fundamental Research Funds for the Central Universities (No. 2021QN028).

## Conflict of interest

The authors declare that the research was conducted in the absence of any commercial or financial relationships that could be construed as a potential conflict of interest.

## Publisher’s note

All claims expressed in this article are solely those of the authors and do not necessarily represent those of their affiliated organizations, or those of the publisher, the editors and the reviewers. Any product that may be evaluated in this article, or claim that may be made by its manufacturer, is not guaranteed or endorsed by the publisher.
