# Supplementary material for: Oral pyruvate prevents high-intensity interval exercise-induced metabolic acidosis in rats by promoting lactate dehydrogenase reaction
Source: Front Nutr. 2023 Apr 6;10:1096986. doi: 10.3389/fnut.2023.1096986 (PMC10117856; doi:10.3389/fnut.2023.1096986)
Supplement: Supplementary file 1 [file Image_1.PDF]

Supplementary Material

Oral pyruvate prevents HIIE-induced metabolic acidosis in rats by promoting LDH reaction

Kaixuan Che, Yanping Yang, Jun Zhang, Lin Feng, Yan Xie, Qinlong Li, Junqiang Qiu*

*** Correspondence:** Junqiang Qiu: qiujunqiang@bsu.edu.cn

1 Supplementary Figures and Tables

1.1 Supplementary Figures

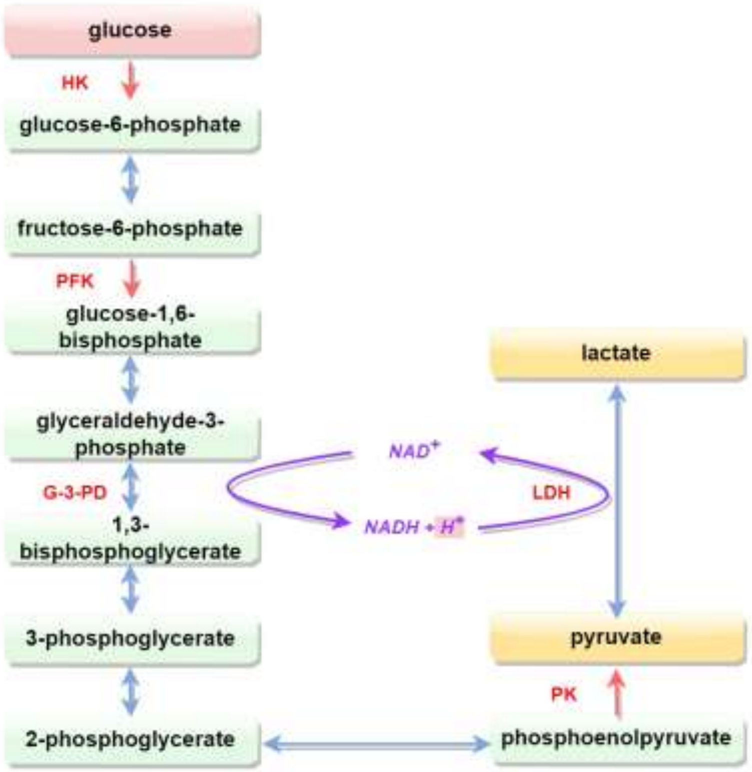

Supplementary Figure 1. Pyruvate anaerobic metabolic pathways and their relationship with the consumption of protons in intracellular compartments. The reduction of pyruvate to lactate with LDH absorbs an H^+ coupled with NADH oxidation to NAD^+ in the cytosol, promoting glycolysis at the G-3-PD step. HK=hexokinase; PFK=phosphofructokinase; G-3-PD=glyceraldehyde-3-phosphate dehydrogenase; PK=pyruvate kinase; LDH=lactate dehydrogenase; NAD^+=oxidised nicotinamide adenine dinucleotide; NADH=reduced nicotinamide adenine dinucleotide.
